# Complete pipeline for Oxford Nanopore Technology amplicon sequencing (ONT‐AmpSeq): from pre‐processing to creating an operational taxonomic unit table

**DOI:** 10.1002/2211-5463.13868

**Published:** 2024-08-07

**Authors:** Patrick Skov Schacksen, Stine Karstenskov Østergaard, Mathias Helmer Eskildsen, Jeppe Lund Nielsen

**Affiliations:** ^1^ Department of Chemistry and Bioscience Aalborg University Aalborg East Denmark

**Keywords:** Amplicon, AmpSeq, Nanopore, OTU table, pipeline, sequencing

## Abstract

Amplicon sequencing has long served as a robust method for characterising microbial communities, and despite inherent resolution limitations, it remains a preferred technique, offering cost‐ and time‐effective insights into bacterial compositions. Here, we introduce ONT‐AmpSeq, a user‐friendly pipeline designed for processing amplicon sequencing data generated from Oxford Nanopore Technology (ONT) devices. Our pipeline enables efficient creation of taxonomically annotated operational taxonomic unit (OTU) tables from ONT sequencing data, with the flexibility to multiplex amplicons on the same barcode. The pipeline encompasses six main steps—statistics, quality filtering, alignment, clustering, polishing, and taxonomic classification—integrating various state‐of‐the‐art software tools. We provide a detailed description of each step, along with performance tests and robustness evaluations using both test data and a ZymoBIOMICS^®^ Microbial Community Standard mock community dataset. Our results demonstrate the ability of ONT‐AmpSeq to effectively process ONT amplicon data, offering valuable insights into microbial community composition. Additionally, we discuss the influence of polishing tools on taxonomic insight and the impact of taxonomic annotation methods on the derived microbial composition. Overall, ONT‐AmpSeq represents a comprehensive solution for analysing ONT amplicon sequencing data, facilitating streamlined and reliable microbial community analysis. The pipeline, along with test data, is freely available for public use.

## Graphical abstract

Introducing ONT‐AmpSeq: a complete pipeline for generating operational taxonomic unit (OTU) tables with taxonomic information from Oxford Nanopore Technology (ONT) amplicon sequencing data. ONT‐AmpSeq offers integrated, user‐defined and customisable features to adjust for amplicon length, multiple amplicons per barcode, sequencing quality, and environment‐specific databases, ensuring optimal resolution. Its user‐friendly interface and efficiency facilitate deep insights into diverse ecosystems and microbial communities.

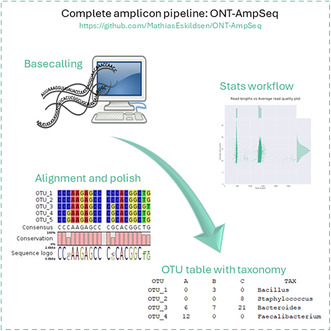

**Abbreviations**
BLAST: Basic Local Alignment Search Tool
ENA: European Nucleotide Archive
FLASV: Full‐length amplicon sequence variant
HPC: High‐performance computing
mcrA: methyl coenzyme‐M reductase
NCBI: National Center for Biotechnology Information
ONT: Oxford Nanopore Technologies
OTU: operational taxonomic unit

Oxford Nanopore Technologies (ONT) has revolutionised sequencing methodologies, offering versatile applications, including amplicon sequencing. While the potential for long‐read sequencing is immense, ONT's main historical challenge lies in the relatively low read quality compared to short‐read sequencing techniques such as Illumina, IonTorrent or Sanger [1]. A significant limitation to these low‐error‐rate sequencing techniques is the relatively short sequencing length, typically ranging from 350 to 800 bp. However, advancements in ONT sequencing technology, such as the introduction of V14 chemistry and R10.4.1 flow cells, have substantially improved sequencing accuracy to Q20+, overcoming the historical limitations of the technique. Continuous improvements in basecalling algorithms and motor protein optimisation are expected to further enhance the quality of ONT reads, with Q28+ simplex reads in the first quarter of 2024 [2]. In addition to the high read quality, the ability to barcode sequences allows simultaneous sequencing of multiple samples. Although other high‐throughput, low‐error‐rate, long‐read sequencing platforms, such as PacBio exist, the higher cost and more labour‐intensive implementation make ONT a more accessible choice. A full‐scale portable sequencing platform from ONT only requires a laptop and an internet connection [3].

A typical workflow for barcoded amplicons includes six steps. First, basecalling and demultiplexing are performed, typically using tools such as dorado (https://github.com/nanoporetech/dorado), while previously performed by the basecaller guppy (https://community.nanoporetech.com), and demultiplexing tools such as porechop [4]. Next, quality filtering is conducted by assessing sequence length and quality with nanoplot [5] and applying subsequent filtering using tools like chopper [6]. The third step involves alignment and clustering to create consensus operational taxonomic units (OTUs) using vsearch [7]. Polishing is then performed to minimise sequence errors from the clustered OTUs using racon [8] or medaka (https://github.com/nanoporetech/medaka). Following this, the OTU table is constructed from the polished OTUs using vsearch. Finally, taxonomic classification based on the consensus OTUs is achieved using either vsearch or blast+ [7, 9], depending on desired database for the project.

Selecting an appropriate combination of up‐to‐date software for each of these steps requires specialised knowledge within the field of bioinformatics. Additionally, these tools are often not designed to be compatible with each other, necessitating correct data handling for proper processing.

Advancements and the versatility of applications for ONT, including short‐ and long‐read sequencing, which present the possibility of developing a standalone pipeline for generating OTU tables. However, currently, there are no commonly available complete workflows that offer integrated amplicon product generation and subsequent taxonomic classification without requiring specialised knowledge of the field. Therefore, the aim of this pipeline is to create a simplified workflow—from barcoded sequences to a taxonomically classified OTU table—incorporating state‐of‐the‐art integrated software and offering versatile customisable utilisation, all in the form of a simple complete package solution.

## Materials and methods

**Fig. 1:**
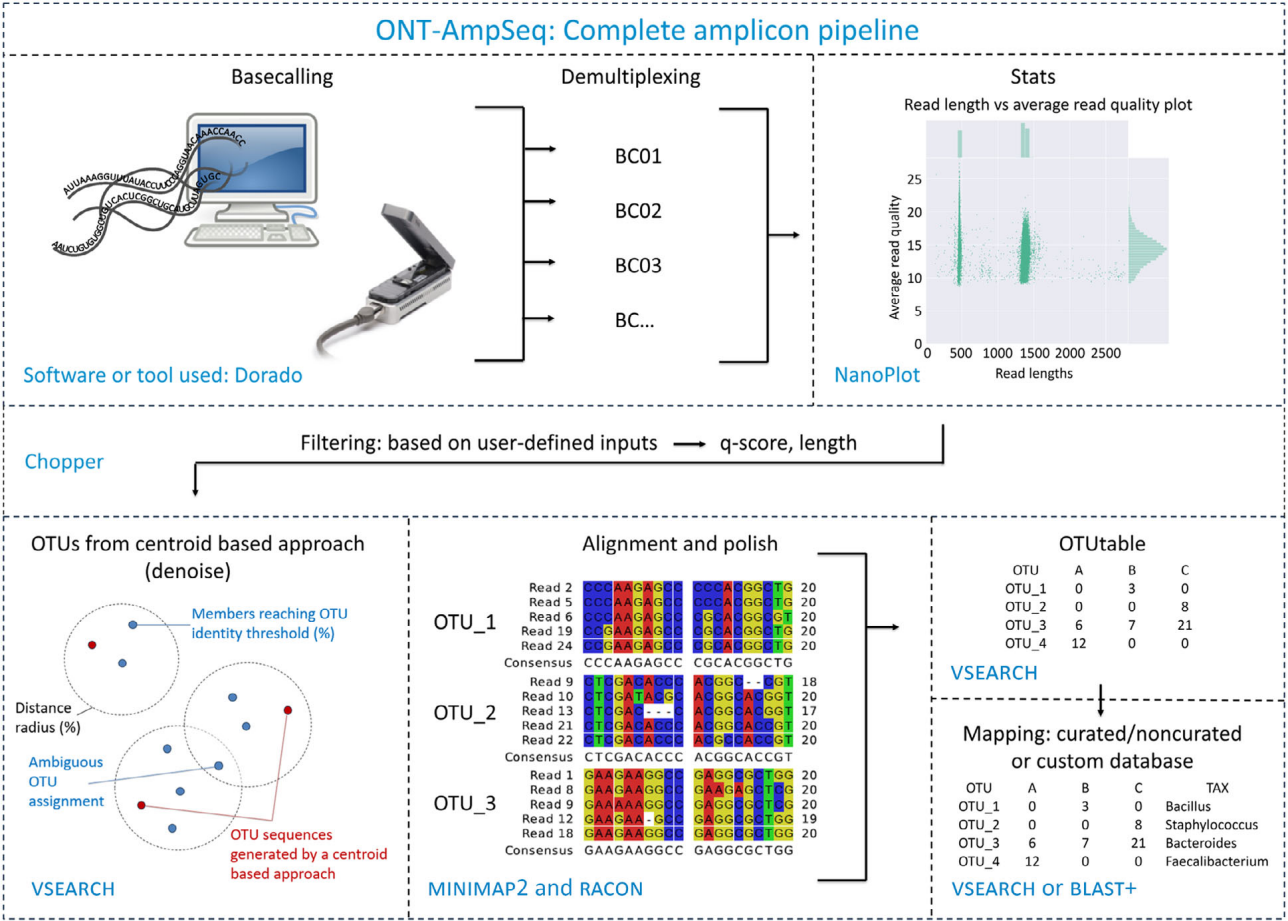
A schematic flowchart of the ONT‐AmpSeq pipeline illustrating the bioinformatic processing of raw reads to the final operational taxonomic units (OTU) table. It outlines the preprocessing of raw sequence data and the six main steps of ONT‐AmpSeq, along with the primary software utilised during each step.

The ONT‐AmpSeq pipeline, developed using snakemake [10], conda, python 3 [11], and bash, integrates a variety of software and tools for processing amplicon sequencing data from ONT sequencing devices. The pipeline consists of two main processes: an initial exploratory “statistics” function and the subsequent “main” pipeline (see Fig. 1 for an overview). The exploratory statistical function generates plots of the user‐defined sequence data, allowing users to assess and set suitable thresholds for amplicon length and quality‐score criteria in the subsequent main script. The main script then generates draft consensus sequences, clusters them into biologically relevant OTUs, creates an OTU table, and annotates taxonomy based on user‐defined databases. The source code is available via GitHub (https://github.com/MathiasEskildsen/ONT‐AmpSeq) and is freely available without the need for a licence. The pipeline is specifically designed for Linux/UNIX shell environment. Dependencies can be installed using anaconda, following instructions provided in the GitHub documentation and the accompanying YAML file, which includes the version history of the tools utilised in the pipeline. Additionally, a comprehensive user manual accessible on GitHub, providing detailed instructions for utilising the pipeline and modifying specific parameters, such as resource allocation and software‐specific settings adjustments. The GitHub also includes a section explaining how to select and download a relevant database for a given project. This section features a user guide for creating a customised database or reformatting an existing database to the correct format, with several examples provided.

### Raw data input

The pipeline is tailored to handle basecalled ONT sequencing data generated, supporting standard fastq output format, in both compressed (“.gz”) and decompressed formats. Basecalling and demultiplexing are typically performed using dorado (https://github.com/nanoporetech/dorado), which is also integrated into the minknow software (https://community.nanoporetech.com), or alternatively through guppy (https://community.nanoporetech.com). In scenarios where reads are basecalled using guppy, demultiplexing is often executed using other tools such as porechop [4] or cutadapt [12]. It is worth mentioning that the pipeline is compatible with demultiplexed data utilising either method.

### Read statistics and filtering

The exploratory statistics function is implemented as a standalone script written in bash. The script utilises nanoplot [5] to generate read statistics and plots for each barcode, offering users a comprehensive overview of amplicon quality and length. The obtained information from this analysis can then be utilised by the user to define specific thresholds for read quality and length. Subsequently, chopper [6] is employed to filter and trim amplicons based on the user‐defined parameters. In cases where a barcode contains multiple amplicons with distinct size differences, users have the option to separate them based on specified length thresholds, thereby generating separate outputs for each amplicon. Allowing users to analyse the microbial composition of multiple amplicons, saving both barcodes and sequencing capacity.

### Generating draft consensuses and OTUs

Draft consensus sequences are produced using vsearch to obtain biologically relevant OTUs [7]. These draft consensus sequences are generated employing a centroid‐based approach, wherein the central sequence of each cluster is designated as the centroid. These centroids are then utilised to generate the draft consensus sequences, which are subsequently denoised to reduce errors. While the centroid‐based approach may eliminate some natural variants during clustering, it ensures accurate identification of biologically significant core OTUs. vsearch was selected as the clustering tool for this pipeline due to its versatility, scalability, and effectiveness in identifying core OTUs.

### Polishing

Despite the rapid advancements in ONT read quality, the implementation of error correction techniques, such as polishing, remains crucial for achieving high‐quality consensus sequences. Various tools are available for this purpose, including racon [8] or ONT's own software, medaka (https://github.com/nanoporetech/medaka), along with a plethora of other polishing tools homopolish [13] or pepper [14]. These tools, among many others, have been utilised either individually or in a combination [15]. To polish with racon, the original filtered reads from chopper are initially mapped to the draft consensus sequences from vsearch through minimap2 [16]. This alignment is then corrected using sequence information from the mapped reads through racon thus generating error corrected OTUs. The efficiency of the polishing process is significantly influenced by the file size used for mapping and can be greatly improved through multithreading or parallelisation of file processing during consensus generation, facilitated by snakemake [10].

### Taxonomic classification and OTU table

The OTUs are subsequently utilised to construct the final OTU table using vsearch, clustering at both 97% and 99% sequence identities for each barcode as a default. The desired sequence identity used for clustering, can easily be modified by the user. Taxonomy is then assigned to the polished consensus OTUs either through a blast+ approach against a blast formatted nucleotide database [9] or via the sintax algorithm [17] using vsearch and a sintax‐formatted fasta database [7]. The blastn method employs an *e*‐value cut‐off at 1*e* ^−10^ by default, which can be adjusted, and provides the complete annotation of the best hit. Conversely, the sintax approach filters taxonomy based on an additional “sintax_cutoff” value, annotating only to a taxonomic level that support the corresponding bootstrap value of the sequence identities. The database utilised for the pipeline is specified through user input and requires a local installation of the desired database in the corresponding format. For optimal annotation, it is recommended to use a manually curated environmentally specific database, such as the mgnify Genomes for the human gut [18] or the 16S rRNA full‐length amplicon sequence variant (FLASV) sintax midas database [19] for 16S rRNA gene from sludge samples or the blast formatted refseq database from ncbi [20] for a more universal annotation of unspecified sample types. These tables, along with their annotated taxonomy, represent the final outputs of the pipeline and can be readily used to visualise the microbial composition of the samples using r packages such as phyloseq [21] and ampvis2 (https://github.com/KasperSkytte/ampvis2), with the sintax based taxonomy annotations preformatted for compatibility with these r packages.

### Test data

The test dataset utilised for assessing the pipeline consist of 10 multiplexed barcoded amplicons samples, encompassing both methyl coenzyme‐M reductase (mcrA) [22] and v1‐8 16S rRNA gene amplicon data on each barcode [23, 24]. The raw test data is accessible at the European Nucleotide Archive (ENA) under the project accession number PRJEB71743, with the corresponding analysed barcodes indicated on GitHub. The ONT‐AmpSeq pipeline demonstrated the ability to effectively segregate multiplexed genes from the same barcode, based on user‐specific filtering inputs. The *mcrA* gene amplicon data were filtered by lengths ranging from 400 to 600 bp and a quality threshold of Q20, then annotated to a blastn‐formatted GenBank database [20]. The 16S rRNA gene data were filtered by lengths ranging from 1200 to 1600 bp and Q20, and subsequently annotated to the sintax‐formatted midas v5.1 database [19]. The workflow and resulting OTU tables can be accessed via the GitHub documentation at https://github.com/MathiasEskildsen/ONT‐AmpSeq.

## Results

### Performance test

A performance test was conducted to evaluate the limitations of the pipeline's capabilities using 16S rRNA gene amplicons from various dataset sizes on a Slurm‐controlled high‐performance computing (HPC) cluster [25] (Table 1). The tests included running the 16S rRNA gene amplicon Zymo data, test data and three additional larger datasets. It was observed that minimap2 was the most memory‐intensive step, with memory usage increasing significantly with larger sizes. The maximum memory utilisation, regardless of single file size or combined dataset size, appeared to plateau at just over 30 GB. Another memory‐intensive step was the generation of draft consensus sequences through vsearch. For example, processing the 1.02 GB single file within the “Large” dataset in Table 1 required 6 GB memory. Regarding CPU hours for different dataset sizes, the number of samples seemed to have a negligible effect, while the total data size notably impacted processing time.

**Table 1:** Performance test of different 16S rRNA gene amplicon datasets. All datasets were all filtered for read length of 1200–1600 bp and different Q‐scores, against the midas 5.1 sintax database. The three other datasets are not shown.

Dataset	Total samples	Dataset size, post‐filtering (GB)	File size, post‐filtering (GB)	Max memory allocated (GB)	Max memory utilised (GB)	CPU hours	Taxonomy
Zymo	3	0.12	0.04–0.05	40	11.6	93	sintax, midas 5.1
Test	10	0.6	0.02–0.17	40	4.1	219	sintax, midas 5.1
Medium	13	2.2	0.11–0.38	40	30.7	5908	sintax, midas 5.1
Medium	20	2.2	0.03–0.39	40	33.4	5113	sintax, midas 5.1
Large	47	4.6	0.01–1.02	42	31	17 240	sintax, midas 5.1

Comparing the taxonomic annotation methods, the sintax algorithm was known to be significantly faster than the blast algorithm, which was also confirmed in a test comparing the two annotation methods. When annotating the v1‐8 test data, the sintax algorithm completed the task in 1 min 15 s, whereas blast took over 2 h. This indicates that sintax was more than 100 times faster without parallelisation. To enhance the speed of blast annotation, this step was optimised to run in parallel using 30 threads, resulting in a processing time of 20 min, representing a six‐fold increase in speed.

### Robustness of the pipeline

**Fig. 2:**
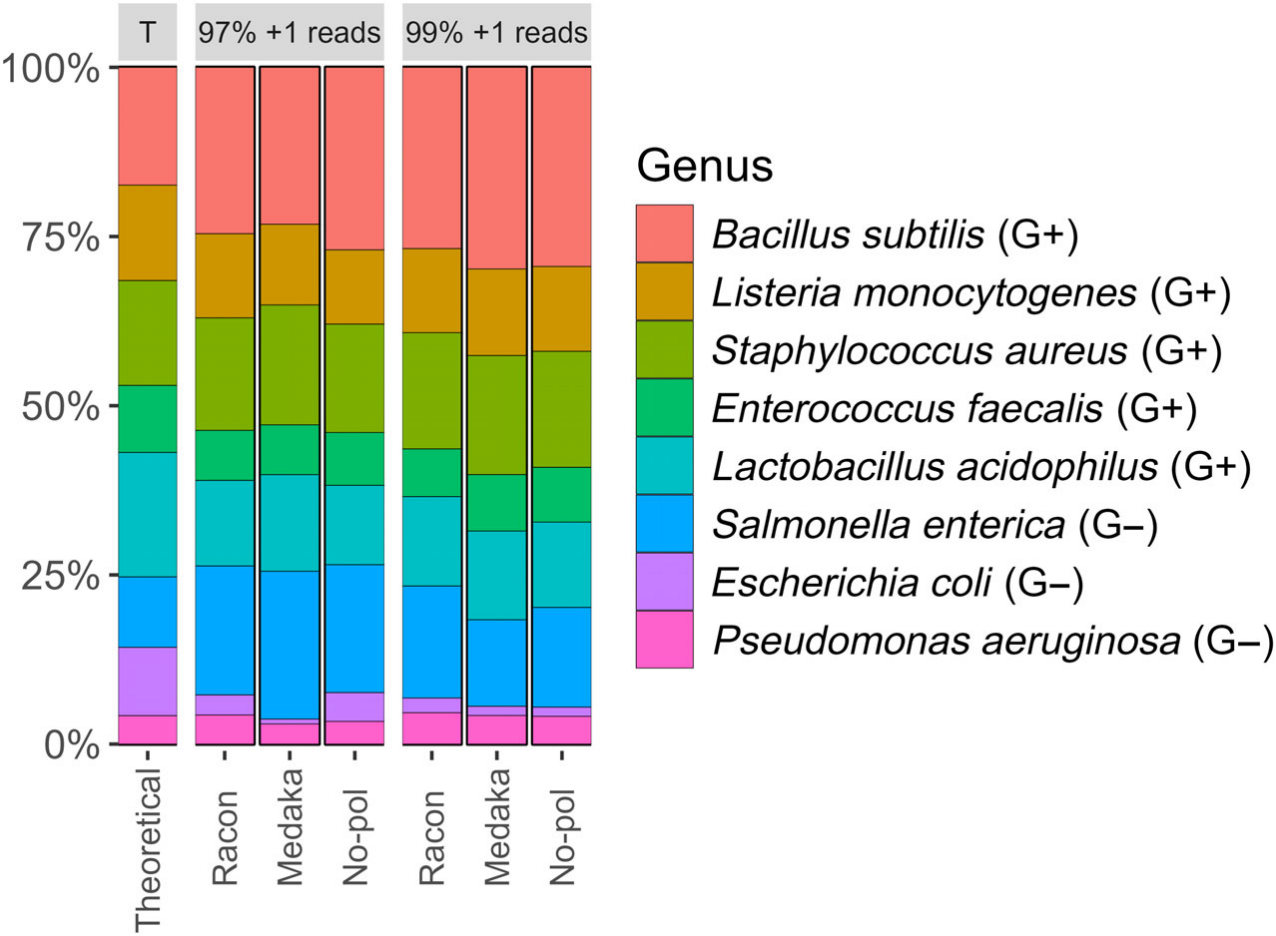
Stacked barplot comparing the theoretical composition of the ZymoBIOMICS® mock data to the test data analysed using ONT‐AmpSeq, employing different clustering identities and polishing tools. T, theoretical composition.

To evaluate the robustness of the ONT‐AmpSeq pipeline, we utilised sequence data from a ZymoBIOMICS^®^ Microbial Community Standard mock community (ncbi BioProject accession number: PRJNA925180). Leveraging this data and the provided theoretical microbial composition alongside reference sequences from Zymo Research, formatted into a custom sintax reference database, we subjected the pipeline to various tests. These tests included assessing the impact of racon and medaka polishing, or lack thereof (no‐pol) (Fig. 2), as well as exploring the effects of clustering at different sequence identities and post‐processing filtering (Fig. S1, Table S1). Our analysis revealed the identification of all eight bacterial genera targeted by the 16S rRNA gene amplicons, which were then compared to the theoretical microbial composition. Interestingly, it was observed that the relative abundance of *Bacillus* and *Salmonella* were consistently more than 5% higher compared to the theoretical composition, while *Escherichia* and *Lactobacillus* were consistently underrepresented by >5%. It is worth noting that factors such as natural variance, PCR bias, or primer selection were not considered in our analysis, which could potentially account for some of the observed variations in relative abundance. Notably, the theoretical composition from ZymoBIOMICS was derived using 16S rRNA gene v3‐4 primers sequenced on Illumina, while the reference data utilised 16S rRNA gene v1‐9 primers and was sequenced using ONT technology.

### Comparing the microbial composition utilising different polishing tools

**Fig. 3:**
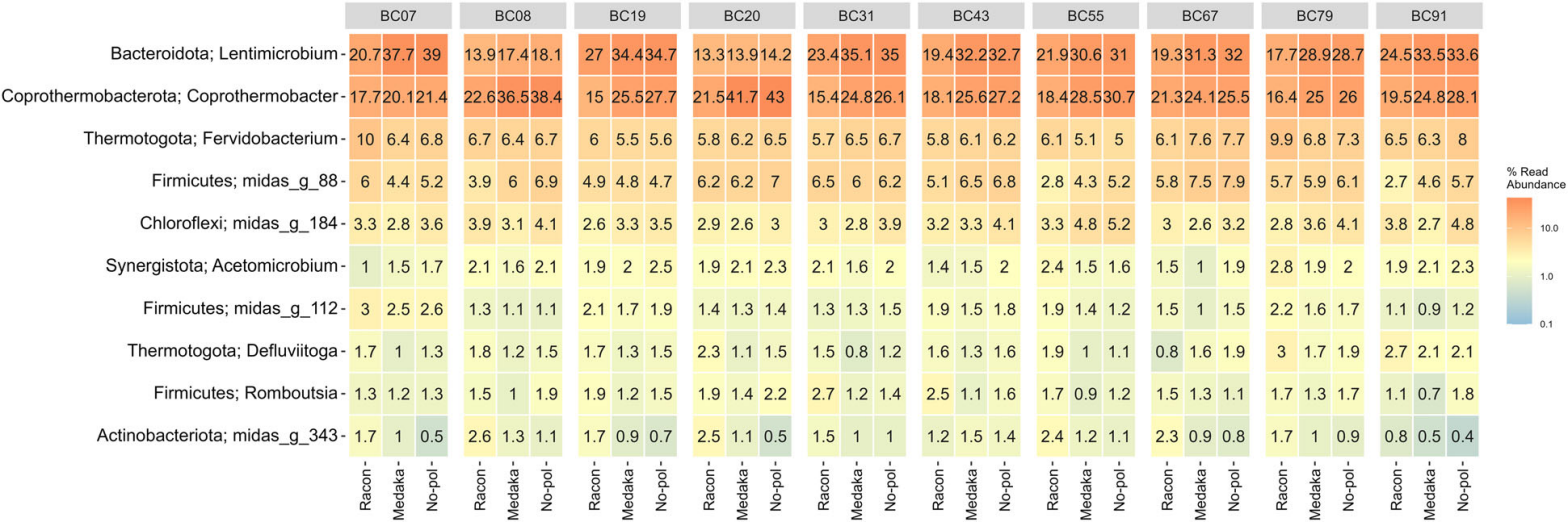
Heatmap depicting the 10 most abundant genera from the v1‐8 16S rRNA gene amplicons test data, clustered at 97% sequence identity and filtered with a + 5 read threshold. Taxonomic annotation was performed using the sintax algorithm to the midas 5.1 database [19].

An assessment of the microbial composition of the 16S rRNA gene test data was conducted, examining the effects of employing different polishing tools and subsequent post‐filtering (Fig. S2). Upon evaluation, it was observed that the microbial composition at genus level using medaka or no polishing exhibited striking similarities at 97% sequence identity (Fig. 3). When comparing the results from racon polishing to those from medaka at 97% sequence identity, racon displayed a lower relative abundance of the two predominant genera, *Lentimicrobium* and *Coprothermobacter*, while the opposite trend was noted for the less abundant genera. Similar trends were observed when comparing the polishing tools at 99% sequence identity without post‐filtering (Fig. S2). However, upon removing singletons at 99% identity, medaka exhibited a similarity to racon rather than no polishing. At this sequence identity, as more reads were filtered, fewer genus annotations were detected using no polishing. Furthermore, upon analysing the number of unique OTUs generated by the difference tools, it was found that medaka and no polishing yielded twice the amount of OTUs compared to racon at 97% sequence identity clustering and approximately 3.5 times more at 99% clustering for this test dataset.

### Comparing blast and sintax annotation

To compare the two taxonomic annotation methods, the racon‐polished 16S rRNA gene test data was mapped to the midas 5.1 flasv sintax database using both annotation methods (Fig. S3). These results revealed clear discrepancies between the annotation methods, particularly with singletons removed. Generally, the blastn annotated taxonomy exhibited higher relative abundances compared to sintax annotation. Notably, certain genera such as *Variibacter* and *Microbacterium* were exclusively identified using blastn, while others like *Romboustia* and *Acetomicrobium* were predominantly annotated using blastn. The discrepancy stemmed from the additional bootstrap support provided at the individual taxonomic level for sintax annotation, leading to certain OTUs lacking genus‐level classification and only being identified up to a higher supported taxonomic rank.

## Discussion

The ONT‐AmpSeq pipeline developed in snakemake provide researchers with a flexible and scalable solution for processing ONT sequencing data, tailored to user‐specific inputs, and is solely constrained by the quality of sequence data and reference database. By automating technical complexities and routine tasks, the pipeline streamlines the analysis process, allowing researchers to focus on interpreting their data and generating meaningful results.

The processing time of the pipeline is, as expected, influenced by the input data size, particularly when dealing with larger datasets and databases during alignment and taxonomic annotation. The performance tests revealed a clear correlation between data size and processing time, with larger datasets requiring proportionally more computational resources. This relationship was further validated through testing various dataset sizes, demonstrating an exponential increase in CPU hours corresponding to the data size. The pipelines scalability is thereby only limited by computational power rather than inherent constraints within the individual tools used. If users provide input data that are appropriately named and preprocessed into demultiplexed individual files, as outlined in the user guide, the pipeline's scalability is primarily constrained by available computational resources.

Based on the findings from both the mock data and test dataset, it is evident that the choice of polishing tool significantly influences the taxonomic insight derived from the datasets. While all polishing tools provided valuable taxonomic information, notable differences and variations in relative abundance were observed among them. Specifically, medaka exhibited a taxonomic distribution more akin to that of the non‐polished dataset.

It is noteworthy that medaka is trained on flye [26], which is optimised for metagenome assembly, while racon primarily focuses on generating genomic consensus. This distinction in functionality may impact the polishing step, considering that the pipeline is designed for amplicon data rather than metagenomic data. These differences in clustering become apparent during dataset filtering and visualisation. Furthermore, the taxonomic annotation results highlight the superiority of the sintax algorithm over the blast algorithm in terms of validity. This superiority stems from the sintax algorithm's incorporation of bootstrap support for each taxonomic rank and its additional filtering based on this support. As a result, sintax provides a more robust taxonomic annotations compared to blast.

### Other software

The concept of generating an OTU table is well‐established, with software like dada2 providing comprehensive solutions for creating tables from Illumina data over the years. However, dada2 is exclusively designed for Illumina data and requires significant user involvement, as all data preprocessing must be manually conducted in r. In contrast, for ONT data, various software and tools exist for generating consensus files, such as ngspecies [27] and amplicon_sorter [28], which have demonstrated the ability to produce consensus sequences comparable to polished medaka reads. However, these tools primarily focus on generating consensus sequences and do not offer OTU table creation or taxonomy annotation functionalities without user‐driven bioinformatic processing knowledge and processing following these. While there are pipelines available for creating OTU tables from ONT data, such as rescue [29], their accuracy without polishing remains untested and is often limited to specific genes and databases. Therefore, while existing tools and pipelines offer some solutions for OTU table generation from ONT data, they lack the robustness and versatility required for comprehensive analysis without additional polishing steps.

## Conclusions

The ONT‐AmpSeq pipeline offers an accessible and comprehensive solution for generating high‐quality, taxonomically annotated OTU tables from standard ONT data. It streamlines the process by allowing users to specify their parameters and sample‐specific databases, eliminating the need for extensive manual configuration. Moreover, it accommodates the sequencing of multiple amplicons under the same barcode, as evidenced by our test data. Another feature of ONT‐AmpSeq is its ability to process any amplicon independent of the sequencing platform, as long as the input format is in fastq format. This versatility extends to any taxonomic kingdom, not limited to prokaryotes but also including eukaryotes, archaea, and even viruses, with the only requirements being amplicon data and a suitable database for the respective annotation. The resulting output is compatible with widely used r packages such as phyloseq and ampvis2, eliminating the need for additional preprocessing steps before downstream analysis and visualisation.

Given the nature of ONT's sequencing data, we advocate for polishing amplicon data using racon due to its efficiency and stability. However, in scenarios where time is a crucial consideration, especially with large datasets comprising numerous barcodes and substantial data exceeding several gigabytes, opting for no polishing can provide valuable insights into biologically relevant OTUs and their relative abundance distribution at 97% sequence identity. Whenever feasible, we advocate for utilising the sintax algorithm for taxonomic annotation, owing to its provision of bootstrap support for each taxonomic level and significantly reduced processing time.

Furthermore, it is worth noting that the pipeline undergoes continuous support and updates on GitHub to stay aligned with advancements in sequencing technologies and developments in bioinformatic software.

## Conflict of interest

The authors declare no conflicts of interest.

### Peer review

The peer review history for this article is available at https://www.webofscience.com/api/gateway/wos/peer‐review/10.1002/2211‐5463.13868.

## Author contributions

PSS contributed to investigation, formal analysis, conceptualisation, data curation, methodology, software, validation, visualisation, writing‐original draft. SKØ contributed to investigation, formal analysis, conceptualisation, data curation, methodology, software, validation, visualisation, writing‐original draft. MHE: formal analysis, conceptualisation, data curation, methodology, software, validation, writing‐original draft. JLN: conceptualisation, writing‐review & editing, funding acquisition, supervision, resources.

## Supporting information


**Fig. S1.** Stacked barplot comparing the theoretical composition of the ZymoBIOMICS^®^ mock data (first column) to the test data analysed using ONT‐AmpSeq, employing different clustering identities, polishing tools and filtering.

**Fig. S2.** Heatmap depicting the 10 most abundant genera from the v1‐8 16S rRNA gene amplicons test data, clustered at 97% and 99% sequence identity and filtered at various read threshold.

**Fig. S3.** Heatmap depicting the 20 most abundant genera from the v1‐8 16S rRNA gene amplicons test data, clustered at 97% sequence identity and filtered with a + 1 read threshold.

**Table S1.** Comparing the sequenced ZymoBIOMICS^®^ mock data (PRJNA925180) to the theoretical bacterial composition in percentage, analysed using ONT‐AmpSeq, employing different clustering identities, polishing tools, and post‐filtering.

## Data Availability

The pipeline and test data are publicly available at https://github.com/MathiasEskildsen/ONT‐AmpSeq. Test amplicon sequencing data is available at the European Nucleotide Archive (ENA) under the project accession number PRJEB71743.
