# Supplementary material for: Complete pipeline for Oxford Nanopore Technology amplicon sequencing (ONT‐AmpSeq): from pre‐processing to creating an operational taxonomic unit table
Source: FEBS Open Bio. 2024 Aug 7;14(11):1779–87. doi: 10.1002/2211-5463.13868 (PMC11532972; doi:10.1002/2211-5463.13868)
Supplement: Supplementary file 1 — Fig. S1. Stacked barplot comparing the theoretical composition of the ZymoBIOMICS® mock data (first column) to the test data analysed using ONT‐AmpSeq, employing different clustering identities, polishing tools and filtering. [file FEB4-14-1779-s002.pdf]

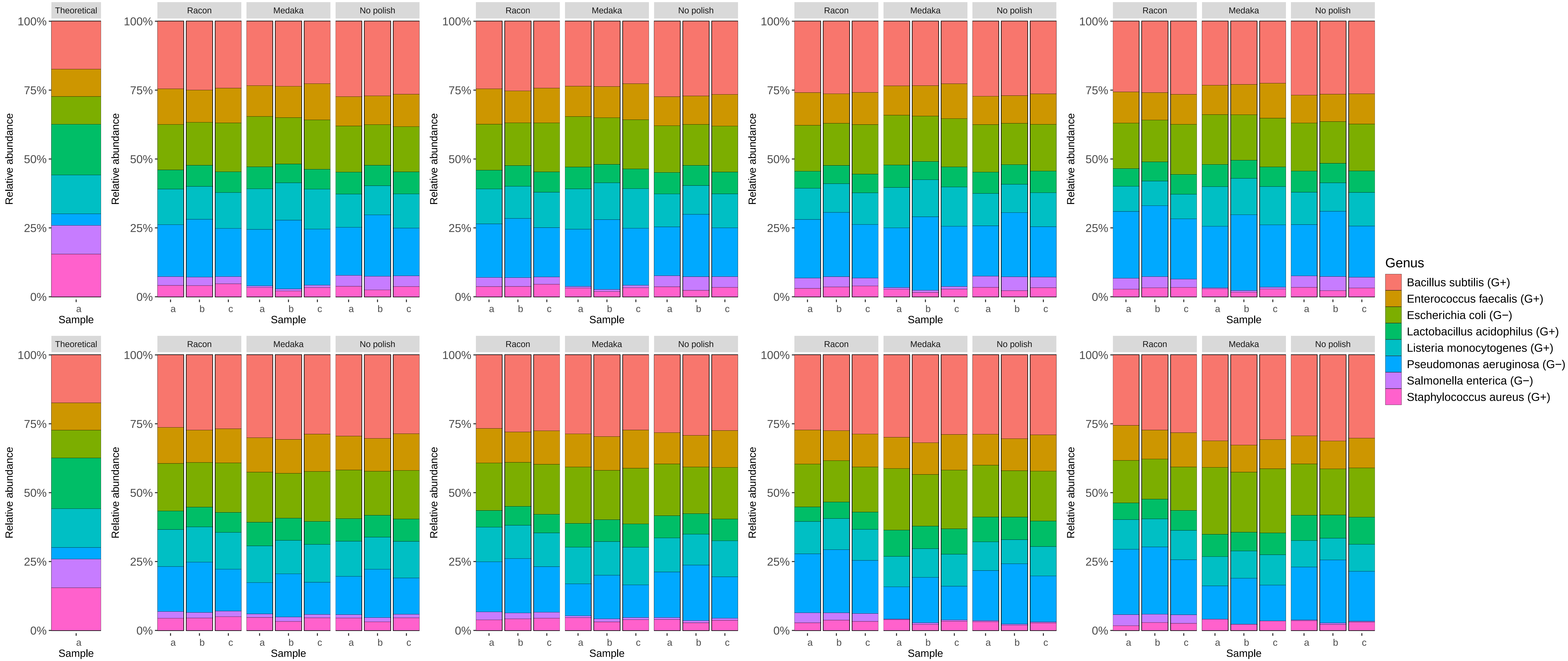

Figure S1: Stacked barplot comparing the theoretical composition of the ZymoBIOMICS® mock data (first column) to the test data analysed using ONT−AmpSeq, employing different clustering identities, polishing tools and filtering.
