# Supplementary material for: Complete pipeline for Oxford Nanopore Technology amplicon sequencing (ONT‐AmpSeq): from pre‐processing to creating an operational taxonomic unit table
Source: FEBS Open Bio. 2024 Aug 7;14(11):1779–87. doi: 10.1002/2211-5463.13868 (PMC11532972; doi:10.1002/2211-5463.13868)
Supplement: Supplementary file 3 — Fig. S3. Heatmap depicting the 20 most abundant genera from the v1‐8 16S rRNA gene amplicons test data, clustered at 97% sequence identity and filtered with a + 1 read threshold. [file FEB4-14-1779-s004.pdf]

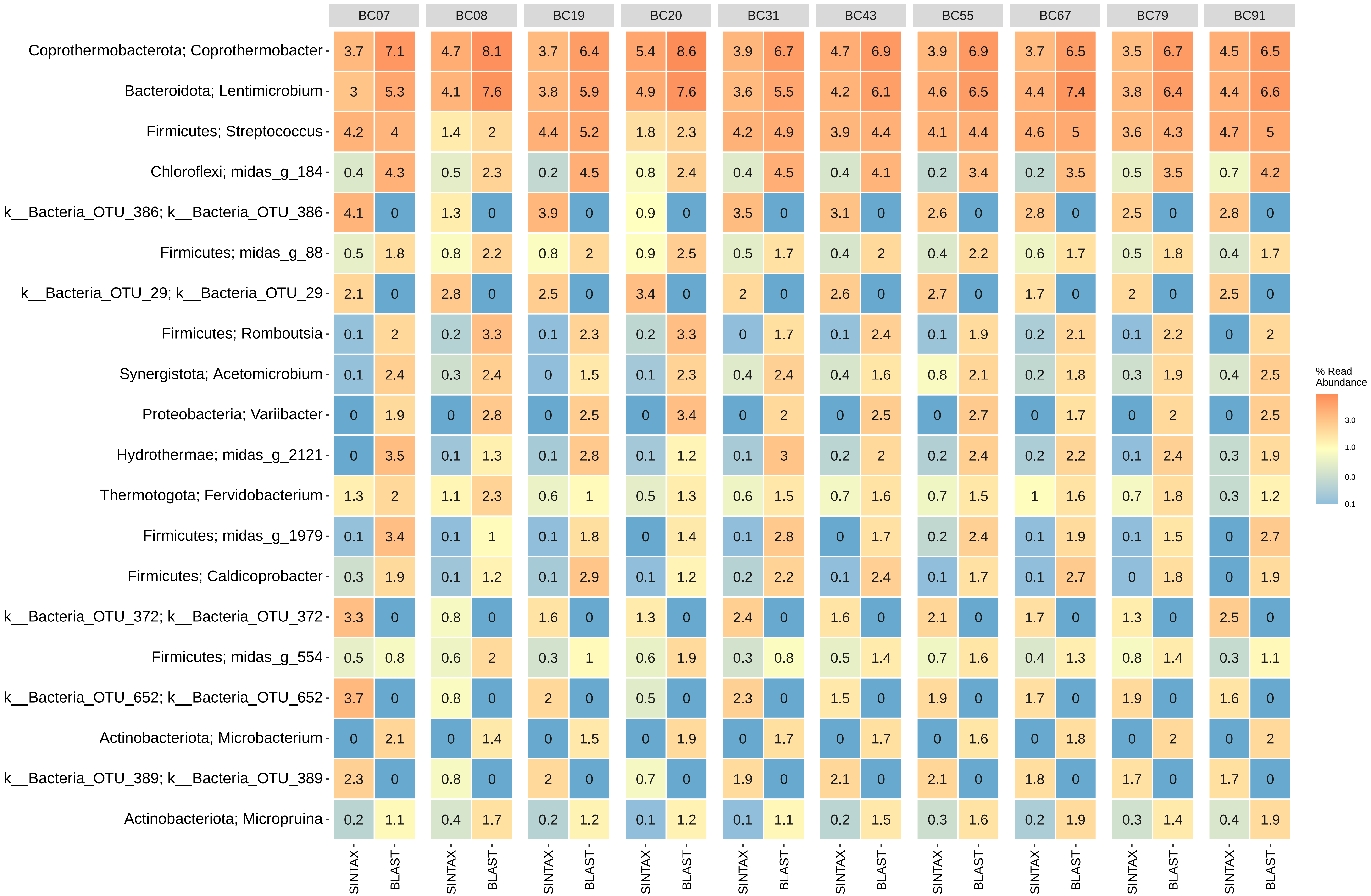

Figure S3: Heatmap depicting the 20 most abundant genera from the v1−8 16S rRNA gene amplicons test data, clustered at 97% sequence identity and filtered with a +1 read threshold.
